# Fractional ridge regression: a fast, interpretable reparameterization of ridge regression

**DOI:** 10.1093/gigascience/giaa133

**Published:** 2020-11-30

**Authors:** Ariel Rokem, Kendrick Kay

**Affiliations:** Department of Psychology and the eScience Institute, University of Washington, Guthrie Hall 119A, Seattle, WA, 98195, USA; Center for Magnetic Resonance Research, University of Minnesota, Twin Cities, 2021 6th St SE, Minneapolis, MN, 55455, USA

**Keywords:** general linear model, hyperparameters, brain imaging, open-source software

## Abstract

**Background:**

Ridge regression is a regularization technique that penalizes the L2-norm of the coefficients in linear regression. One of the challenges of using ridge regression is the need to set a hyperparameter (α) that controls the amount of regularization. Cross-validation is typically used to select the best α from a set of candidates. However, efficient and appropriate selection of α can be challenging. This becomes prohibitive when large amounts of data are analyzed. Because the selected α depends on the scale of the data and correlations across predictors, it is also not straightforwardly interpretable.

**Results:**

The present work addresses these challenges through a novel approach to ridge regression. We propose to reparameterize ridge regression in terms of the ratio γ between the L2-norms of the regularized and unregularized coefficients. We provide an algorithm that efficiently implements this approach, called fractional ridge regression, as well as open-source software implementations in Python and matlab (https://github.com/nrdg/fracridge). We show that the proposed method is fast and scalable for large-scale data problems. In brain imaging data, we demonstrate that this approach delivers results that are straightforward to interpret and compare across models and datasets.

**Conclusion:**

Fractional ridge regression has several benefits: the solutions obtained for different γ are guaranteed to vary, guarding against wasted calculations; and automatically span the relevant range of regularization, avoiding the need for arduous manual exploration. These properties make fractional ridge regression particularly suitable for analysis of large complex datasets.

## Introduction

Consider the standard linear model setting *Y* = *X*β solved for β, where *Y* is a data matrix of dimensionality *d* by *t* (*d* data points in each of *t* targets), *X* is the design matrix with dimensionality *d* by *p* (*d* data points for each of *p* predictors), and β is a coefficient matrix with dimensionality *p* by *t* (with *p* coefficients, one for each predictor, for each of the targets). Ordinary least-squares regression (OLS) and regression based on the Moore-Penrose pseudoinverse (in cases where *p* > *d*) attempt to find the set of coefficients β that minimize squared error for each of the targets *y*. While these unregularized approaches have some desirable properties, in practical applications where noise is present, they tend to overfit the coefficient parameters to the noise present in the data. Moreover, they tend to cause unstable parameter estimates in situations where predictors are highly correlated.

Ridge regression [1] addresses these issues by trading off the addition of some bias for the reduction of eventual error (e.g., measured using cross-validation [2,3]). It does so by not only penalizing the sum of the squared errors in fitting the data for each target but by also minimizing the squared L2-norm of the solution, $||\beta ||_2^2 = \sum {(\beta ^2)}$. Fortunately, this form of regularization does not incur a substantial computational cost. This is because it can be implemented using the same numerical approach for solving unregularized regression, with the simple addition of a diagonal matrix α*I* to the standard matrix equations. Thus, the computational cost of solving ridge regression is essentially identical to that of the unregularized solution. Thanks to its simplicity, computational expedience, and its robustness in different data regimes, ridge regression is a popular technique, with the classic references describing the method [1,4] cited >25,000 times according to Google Scholar.

However, beneath the apparent simplicity of ridge regression is the fact that for most applications, it is impossible to determine *a priori* the degree of regularization that yields the best solution. This means that in typical practice, researchers must test several different hyperparameter values α and select the one that yields the least cross-validation error on a set of data specifically held out for hyperparameter selection. In large-scale data problems, the number of data points *d*, number of predictors *p*, and/or number of targets *t* can be quite large. This has the consequence that the number of hyperparameter values that are tested, *f*, can pose a prohibitive computational barrier.

Given the difficulty of predicting the effect of α on solution outcomes, it is common practice to test values that are widely distributed on a log scale (e.g., see [5]). Although this approach is not grounded in a particular theory, as long as the values span a large enough range and are spaced densely enough, an approximate minimum of the cross-validation error is likely to be found. But testing many α values can be quite costly, and the practitioner might feel tempted to cull the set of values tested. In addition, it is always a possibility that the initial chosen range might be mismatched to the problem at hand. Sampling α values that are too high or too low will produce non-informative candidate solutions that are either over-regularized (α too high) or too similar to the unregularized solution (α too low). Thus, in practice, conventional implementations of ridge regression may produce poor solutions and/or waste substantial computational time.

Here, we propose a simple reparameterization of ridge regression that overcomes the aforementioned challenges. Our approach is to produce coefficient solutions that have an L2-norm that is a pre-specified fraction of the L2-norm of the unregularized solution. In this approach, called “fractional ridge regression" (FRR), redundancies in candidate solutions are avoided because solutions with different fractional L2-norms are guaranteed to be different. Moreover, by targeting fractional L2-norms that span the full range from 0 to 1, the FRR approach explores the full range of effects of regularization on β values from under- to over-regularization, thus ensuring that the best possible solution is within the range of solutions explored. We provide a fast and automated algorithm to calculate FRR, and provide open-source software implementations in Python and matlab. We demonstrate in benchmarking simulations that FRR is computationally efficient for even extremely large data problems, and we show that FRR can be applied successfully to real-world data and delivers clear and interpretable results. Overall, FRR may prove particularly useful for researchers tackling large-scale datasets where automation, efficiency, and interpretability are critical.

## Methods

### Background and theory

Consider the dataset *Y* with dimensionality *d* (number of data points) by *t* (number of targets). Each column in *Y* represents a separate target for linear regression:

(1) \begin{equation*}
y = X\beta + \epsilon, \end{equation*}

where *y* is the measured data for a single target (dimensionality *d* by 1), *X* is the “design” matrix with predictors (dimensionality *d* by *p*), β are the coefficients (dimensionality *p* by 1), and ϵ is a noise term. Our typical objective is to solve for β in a way that minimizes the squared error. If *X* is full rank, the OLS solution to this problem is

(2) \begin{equation*}
\hat{\beta }^{\mathrm{OLS}} = (X^{\intercal } X)^{-1} X^{\intercal } y, \end{equation*}

where *X*^⊺^ is the transpose of *X*. This solution optimally finds the values of β that provide the minimal sum-of-squared error on the data: ∑(*y* − *X*β)^2^. In cases where *X* is not full rank, the OLS solution is no longer well defined and the Moore-Penrose pseudoinverse is used instead. We refer to these unregularized approaches collectively as OLS.

To regularize the OLS solution, ridge regression applies a penalty (α) to the squared L2-norm of the coefficients, leading to a different estimator for β:

(3) \begin{equation*}
\hat{\beta }^{\mathrm{RR}} = (X^{\intercal } X + \alpha I)^{-1} X^{\intercal } y, \end{equation*}

where α is a hyperparameter and *I* is the identity matrix [1,4]. For computational efficiency, it is well known that the original problem can be rewritten using singular value decomposition (SVD) of the matrix *X* [6]:

(4) \begin{equation*}
X = U S V^{\intercal }
\end{equation*}

with *U* having dimensionality *d* by *p, S* having dimensionality *p* by *p*, and *V* having dimensionality *p* by *p*.

Note that *S* is a square matrix:

\begin{equation*} S= \left[ {\begin{array}{ccccc}\lambda _1 & 0 & ... & \\ 0 & \lambda _2 & 0 & ... \\ 0 & 0 & \lambda _3 & 0 & ... \\ \vdots \\ ... & 0 & 0 & 0 & \lambda _p \\ \end{array} } \right]
\end{equation*}

with λ_*i*_ as the singular values ordered from largest to smallest. Replacing the design matrix *X* with its SVD, we obtain

(5) \begin{equation*}
y = U S V^{\intercal } \beta + \epsilon . \end{equation*}

Given that *U* and *V* are unitary (e.g., *U*^⊺^*U* is *I*), left-multiplying each side with *U*^⊺^ produces

(6) \begin{equation*}
U^{\intercal } y = SV^{\intercal }\beta + U^{\intercal }\epsilon . \end{equation*}

Let $\tilde{y} = U^ty$, $\tilde{\beta } = V^{\intercal }\beta$, and $\tilde{\epsilon } = U^t\epsilon$. These are transformations (rotations) of the original quantities (*y*, β, and ϵ) through the unitary matrices *U*^*t*^ and *V*^*t*^. In cases where *p* < *d*, this also projects the quantities into a lower-dimensional space of dimensionality *p*. The OLS solution can be obtained in this space:

(7) \begin{equation*}
\tilde{\hat{\beta }}^{\mathrm{OLS}} = (S^{\intercal }S)^{-1} S^{\intercal }\tilde{y}, \end{equation*}

which simplifies to the following:

(8) \begin{equation*}
\tilde{\hat{\beta }}^{\mathrm{OLS}} = S^{-2} (S^{\intercal } \tilde{y}), \end{equation*}

where

\begin{equation*} S^{-2}= \left[ {\begin{array}{ccccc}\frac{1}{\lambda _1^2} & 0 & ... & \\ 0 & \frac{1}{\lambda _2^2} & 0 & ... \\ 0 & 0 & \frac{1}{\lambda _3^2} & 0 & ... \\ \vdots \\ ... & 0 & 0 & 0 & \frac{1}{\lambda _p^2}\\ \end{array} } \right]
\end{equation*}

is the inverse of the square of the singular value matrix *S*. Thus, for a single coordinate *i* in the lower-dimensional space, we can solve the OLS problem with a scalar multiplication:

(9) \begin{equation*}
\tilde{\hat{\beta }}^{\mathrm{OLS}}_i = \frac{1}{\lambda _i^2} \lambda _i \tilde{y_i}, \end{equation*}

which simplifies finally to

(10) \begin{equation*}
\tilde{\hat{\beta }}^{\mathrm{OLS}}_i = \frac{\tilde{y_i}}{\lambda _i}. \end{equation*}

The SVD-based reformulation of regression described above is additionally useful because it provides insight into the nature of ridge regression [7]. Specifically, consider the ridge regression solution in the low-dimensional space:

(11) \begin{equation*}
\tilde{\hat{\beta }}^{\mathrm{RR}} = (S^{\intercal }S + \alpha I)^{-1} S^{\intercal }\tilde{y}. \end{equation*}

To compute this solution, we note that:

(12) \begin{equation*}
S^tS + \alpha I = \left[ {\begin{array}{ccccc}\lambda _1^2 + \alpha & 0 & ... & \\ 0 & \lambda _2^2 + \alpha & 0 & ... \\ 0 & 0 & \lambda _3^2 + \alpha & 0 & ... \\ \vdots \\ ... & 0 & 0 & 0 & \lambda _p^2 + \alpha \\ \end{array} } \right] , \end{equation*}

the inverse of which is

(13) \begin{equation*} (S^tS + \alpha I)^{-1} = \left[ {\begin{array}{ccccc}\frac{1}{\lambda _1^2 + \alpha } & 0 & ... & \\ 0 & \frac{1}{\lambda _2^2 + \alpha } & 0 & ... \\ 0 & 0 & \frac{1}{\lambda _3^2 + \alpha }& 0 & ... \\ \vdots \\ ... & 0 & 0 & 0 & \frac{1}{\lambda _p^2 + \alpha } \\ \end{array} } \right] . \end{equation*}

Finally, plugging into equation 11, we obtain

(14) \begin{equation*}
\tilde{\hat{\beta }}^{\mathrm{RR}}_i = \frac{\lambda _i}{\lambda _i^2 + \alpha } \tilde{y_i} . \end{equation*}

This shows that in the low-dimensional space, ridge regression can be solved using scalar operations.

To further illustrate the relationship between the ridge regression and OLS solutions, by plugging equation 10 into equation 14, we observe the following:

(15) \begin{equation*}
\tilde{\hat{\beta }}^{\mathrm{RR}}_i = \frac{\lambda _i^2}{\lambda _i^2 + \alpha } \tilde{\hat{\beta _i}}^{\mathrm{OLS}} . \end{equation*}

In other words, the ridge regression coefficients are simply scaled-down versions of the OLS coefficients, with a different amount of shrinkage for each coefficient. Coefficients associated with larger singular values are less shrunken than those with smaller singular values.

To obtain solutions in the original space, we left-multiply the coefficients with *V*:

(16) \begin{equation*}
\hat{\beta } = V\tilde{\hat{\beta }} . \end{equation*}

We now turn to FRR. The core concept of FRR is to reparameterize ridge regression in terms of the amount of shrinkage applied to the overall L2-norm of the solution. Specifically, we define the fraction γ as follows:

(17) \begin{equation*}
\gamma = \frac{||\tilde{\hat{\beta }}^{\mathrm{RR}}||_2}{||\tilde{\hat{\beta }}^{\mathrm{OLS}}||_2} . \end{equation*}

Because *V* is a unitary transformation, the L2-norm of a coefficient solution in the low-dimensional space, $||\hat{\tilde{\beta }}||_2$, is identical to the L2-norm of the coefficient solution in the original space, $||\hat{\beta }||_2$. Thus, we can operate fully within the low-dimensional space and be guaranteed that the fractions will be maintained in the original space.

In FRR, instead of specifying desired values for α, we instead specify values of γ between 1 (no regularization) and 0 (full regularization, corresponding to shrinking all the coefficients to β = 0). But how can one compute the ridge regression solution for a specific desired value of γ? Based on equations 9 and 14, it is easy to calculate the value of γ corresponding to a specific given α value:

(18) \begin{equation*}
\gamma = \frac{||\tilde{\hat{\beta }}^{RR}||_2}{||\tilde{\hat{\beta }}^{OLS}||_2} = \sqrt{\frac{\sum {\left[\lambda _i \tilde{y}_i/\left(\lambda _i^2 + \alpha \right)\right]^2}}{\sum {(\tilde{y}_i/\lambda _i})^2}} . \end{equation*}

In some special cases, this calculation can be considerably simplified. For example, if the singular value spectrum of *X* is flat (λ_*i*_ = λ_*j*_ for any *i* ≠ *j*), we can set all the singular values to λ, yielding the following:

(19) \begin{equation*}
\gamma = \sqrt{\frac{ [\lambda/\left(\lambda ^2 + \alpha \right)]^2 {\sum {\tilde{y}^2_i}}}{ (1/\lambda)^2 \sum {\tilde{y_i}^2 }}} = \frac{\lambda/\left(\lambda ^2 + \alpha \right) {}}{1/\lambda } = \frac{\lambda ^2}{\lambda ^2 + \alpha }. \end{equation*}

This recapitulates the result obtained in Hoerl and Kennard [1], equation 2.6. We can then solve for α:

(20) \begin{equation*}
\alpha = \lambda ^2 \left(\frac{1}{\gamma } - 1\right) . \end{equation*}

Thus, in this case, there is an analytic solution for the appropriate α value, and one can proceed to compute the ridge regression solution using equation 14.

Another special case is if we assume that the absolute values of $\tilde{\beta }_i^{\mathrm{OLS}}$ are all the same. In this case, we can use a few simplifications to calculate the shrinkage in terms of L1-norm:

(21) \begin{equation*} \begin{split} \frac{||\tilde{\hat{\beta }}^{\mathrm{RR}}||_1}{||\tilde{\hat{\beta }}^{\mathrm{OLS}}||_1} & = \frac{\sum {\left| \left(\lambda _i^2 \tilde{\hat{\beta _i}}^{\mathrm{OLS}}\right)/\left(\lambda _i^2 + \alpha \right) \right|}}{\sum {\left|\tilde{\hat{\beta _i}}^{\mathrm{OLS}} \right|}} \\ = \frac{\sum {\left|\left[\lambda _i^2 (\tilde{y_i}/\lambda _i)\right]/\left(\lambda _i^2 + \alpha \right) \right|}}{\sum {\left|\tilde{y_i}/\lambda _i \right|}} & = \frac{\sum {\left(\lambda _i^2 \left|\tilde{y_i}/\lambda _i \right|\right)/\left(\lambda _i^2 + \alpha \right)}}{\sum {\left|\tilde{y_i}/\lambda _i \right|}} \\ & = \frac{\sum {\lambda _i^2/\left(\lambda _i^2 + \alpha \right)}}{p} \end{split}
\end{equation*}

Note that this is the average of the shrinkages for individual coefficients from equation 15. The sum of these shrinkages (this quantity multiplied by *p*):

(22) \begin{equation*}
\sum {\frac{\lambda _i^2}{\lambda _i^2 + \alpha }}
\end{equation*}

has previously been defined as the effective degrees of freedom of ridge regression (See [8], pg. 68). Note that the L1-norm here refers to the rotated space and may not be identical to the L1-norm in the original space.

These two special cases have the appealing feature that the regularization level can be controlled on the basis of examining only the design matrix *X*. However, they rely on strong assumptions that are not guaranteed to hold in general. Thus, for accurate ridge regression outcomes, we see no choice but to develop an algorithm that uses both the design matrix *X* and the data values *y*.

### Algorithm

Our proposed algorithm for solving FRR is straightforward: it evaluates γ for a range of α values and uses interpolation to determine the α value that achieves the desired fraction γ. Although this method relies on brute force and may not seem mathematically elegant, it achieves accurate outcomes and, somewhat surprisingly, can be carried out with minimal computational cost.

The algorithm receives as input a design matrix *X*, target variables *Y*, and a set of requested fractions γ. The algorithm calculates the FRR solutions for all targets in *Y*, returning estimates of the coefficients $\hat{\beta }$ as well as the values of hyperparameter α that correspond to each requested γ. In the text below, we indicate the lines of code that implement each step of the algorithm in the matlab (designated with “M”) and Python (designated with “P”) implementations, where line numbers refer to version 1.2 of the software, available to download at: https://github.com/nrdg/fracridge/releases/tag/1.2:

1. Compute the SVD of the design matrix, *USV*^⊺^ = *X* (M251, P151). To avoid numerical instability, very small singular values of *X* are treated as 0.
2. The data are transformed $\tilde{y} = U^{\intercal }y$ (M258, P62).
3. The OLS problem is solved with one broadcast division operation (equation 10) (M276, P64).
4. The values of α that correspond to the requested γ value are within a range that depends on the singular values of *X* (by equation 18). A series of initial candidate values of α are selected to span a log-spaced range from $10^{-3} \lambda _p^2$, much smaller than the smallest singular value of the design matrix, to $10^3 \lambda _1^2$, much larger than the largest singular value of the design matrix (M302, P165–168). On the basis of testing on a variety of regression problems, we settled on a spacing of 0.2 log_10_ units within the range of candidate α values. This provides fine enough gridding such that interpolation results are nearly perfect (empirical fractions are ∼1% or less from the desired fractions).
5. Based on equation 15, a scaling factor for every value of α and every singular value λ is calculated as (M316, P173):
  (23) \begin{equation*}
\mathrm{SF}_{i, j} = \lambda _i^2 / (\lambda _i^2 + \alpha _j) . \end{equation*}
6. The main loop of the algorithm iterates over targets. For every target, the scaling in equation 23 is applied to the computed OLS coefficients (from Step 3), and the L2-norm of the solution at each α_*j*_ is divided by the L2-norm of the OLS solution to determine the fractional length, γ_*j*_ (M336–349, P188–191). Because the relationship between α and γ may be different for each target, the algorithm requires looping over targets and cannot take advantage of broadcasting across targets.
7. Interpolation is used with α_*j*_ and γ_*j*_ to find values of α that correspond to the desired values of γ (M367, P194). These target α values are then used to calculate the ridge regression solutions via equation 15 (M373, P203).
8. After the iteration over targets is complete, the solutions are transformed to the original space by multiplying $\hat{\beta } = V \tilde{\hat{\beta }}$ (M422, P207).

In terms of performance, this algorithm requires just one (potentially computationally expensive) initial SVD of the design matrix. Operations performed on a per-target basis are generally inexpensive, relying on fast vectorized array operations, with the exception of the interpolation step. Although a large range of candidate α values are evaluated internally by the algorithm, these values are eventually discarded, thereby avoiding costs associated with the final step (multiplication with *V*).

### Software implementation

**Figure 1:**
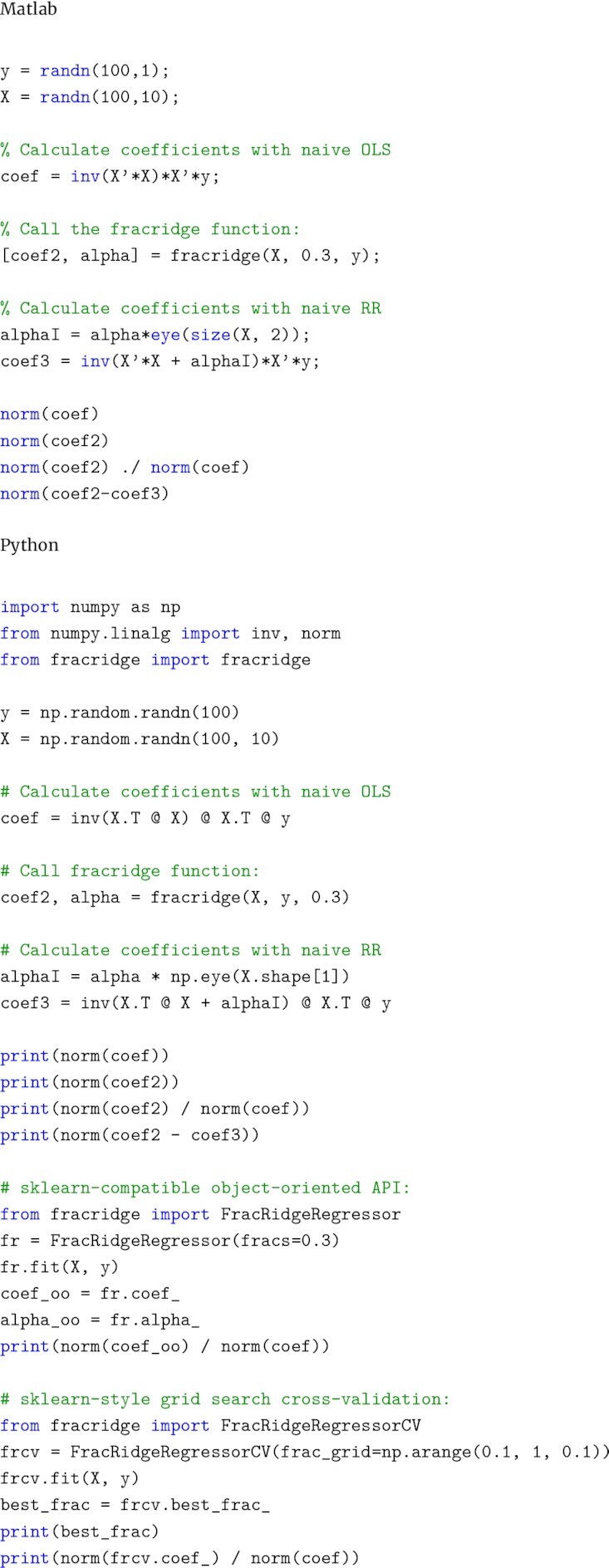
Code examples. Top: MATLAB examples that demonstrate the software API and correctness of the implementation. Bottom: Python examples demonstrate a similar API and correctness. Python examples include the Scikit-Learn-compatible API.

We implemented the algorithm described in Section Algorithm in two different popular statistical computing languages: matlab and Python (example code in Fig. 1). The code for both implementations is available at the project home page (https://nrdg.github.io/fracridge) and released under an OSI-approved, permissive open-source license to facilitate its broad use. In both matlab and Python, we used broadcasting to rapidly perform computations over multiple dimensions of arrays.

There are two potential performance bottlenecks in the code. One is the SVD step, which is expensive in terms of both memory and computation time. In the case where *d* < *p* (the number of data points is smaller than the number of parameters), the number of singular values is set by *d*. In the case where *d* > *p* (the number of data points is larger than the number of parameters), the number of singular values is set by *p*, and our implementation exploits the fact that we can replace the singular values of *X* by the square roots of the singular values of *X*^⊺^*X*, which is only *p* by *p*. This optimization requires less memory for the SVD computation than an SVD of the full matrix *X*. The other potential performance bottleneck is the interpolation performed for each target. To optimize this step, we used fast interpolation functions that assume sorted inputs.

#### MATLAB

The matlab implementation of FRR relies only on core matlab functions and a fast implementation of linear interpolation [9], which is copied into the FRACRIDGE source code, together with its license, which is compatible with the FRACRIDGE license. The matlab implementation includes an option to automatically standardize predictors (either center or also scale the predictors) before regularization if desired.

#### Python

The Python implementation of FRR depends on Scipy [10] and Numpy [11]. The object-oriented interface provided conforms with the API of the popular Scikit-Learn library [12,13], including automated tests that verify compliance with this API (using Scikit Learn’s `check_estimator` function, which automatically confirms this compliance). In addition to an estimator that fits FRR, a cross-validation object is implemented, using Scikit Learn’s grid-search cross-validation API. Unit tests are implemented using pytest [14]. Documentation is automatically compiled using sphinx, with sphinx-gallery examples [15]. The Python implementation also optionally uses Numba [16] for just-in-time compilation of a few of the underlying numerical routines used in the implementation. This functionality relies on an implementation provided in the hyperlearn library [17] and copied into the FRACRIDGE source code, together with its license, which is compatible with the FRACRIDGE license. In addition to its release on GitHub, the software is available to install through the Python Package Index (PyPI) through the standard Python Package Installer (pip install fracridge). For Python, we did not implement standardization procedures because those are implemented as a part of Scikit-Learn.

### Simulations

**Figure 2:**
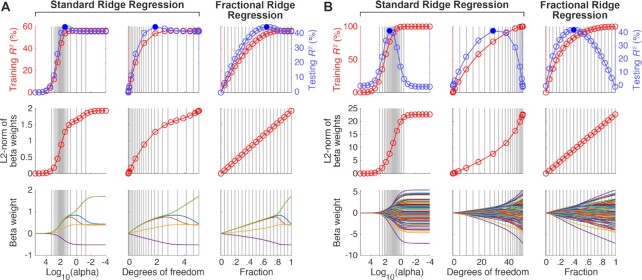
Fractional ridge regression (FRR) achieves desired outcomes. (A) Example regression scenario (*d* = 100, *p* = 5). The first 2 columns show the results of standard ridge regression in which log-spaced α values are used to obtain different levels of regularization. Whereas the first column shows results as a function of log_10_(α), the second column shows results as a function of α values converted to effective degrees of freedom (see Methods). The third column shows the results of FRR in which different regularization levels are achieved by requesting specific fractional L2-norm (γ). Solid blue dots mark peak cross-validation performance. Vertical gray lines in the third column indicate regression solutions obtained by the FRR method (requested fractions range from 0 to 1 in increments of 0.05). The corresponding locations of these regression solutions in the first and second columns are also shown using vertical gray lines. The bottom row shows coefficient paths, i.e., the values of β as a function of log_10_(α), degrees of freedom, or fraction γ. (B) Example regression scenario (*d* = 100, *p* = 100). Same format as panel A. Note that in both scenarios, only the FRR method achieves regression solutions whose L2-norms increase linearly, with gradually changing coefficient paths.

Numerical simulations were used to characterize FRR and compare it to a heuristic approach for hyperparameter selection. Simulations were conducted using the MATLAB implementation (Figure 2). We simulated two simple regression scenarios. The number of data points (*d*) was 100, and the number of predictors (*p*) was either 5 or 100. In each simulation, we first created a design matrix X (*d, p*) using the following procedure: (i) generate normally distributed values for X, (ii) induce correlation between predictors by selecting two predictors at random, setting one of the predictors to the sum of the two predictors plus normally distributed noise, and repeating this procedure 2*p* times, and (iii) *z*-scoring each predictor. Next, we created a set of “ground truth” coefficients β with dimensions (*p*, 1) by drawing values from the normal distribution. Finally, we simulated responses from the model (*y* = *X*β) and added normally distributed noise, producing a target variable *y* with dimensions (*d*, 1).

Given design matrix *X* and target *y*, cross-validated regression was carried out. This was done by splitting *X* and *y* into two halves (50/50 training/testing split), solving ridge regression on one half (training) and evaluating generalization performance of the estimated regression β weights on the other half (testing). Performance was quantified using the coefficient of determination (*R*^2^). For standard ridge regression (SRR), we evaluated a grid of α values that included 0 and ranged from 10^−4^ through 10^5.5^ in increments of 0.5 log_10_ units. For FRR, we evaluated a range of fractions γ from 0 to 1 in increments of 0.05. Thus, the number of hyperparameter values was *f* = 21 in both cases.

The code that implements these simulations is available in the “examples” folder of the software.

### Performance benchmark

To characterize the performance of the FRR and SRR approaches, a set of numerical benchmarks was conducted using the MATLAB implementation. A range of regression scenarios were constructed. In each experiment, we first constructed a design matrix X (*d, p*) consisting of values drawn from a normal distribution. We then created “ground truth” coefficients β (*p, t*) also by drawing values from the normal distribution. Finally, we generated a set of data *Y* (*d, t*) by predicting the model response (*y* = *X*β) and adding zero-mean Gaussian noise with standard deviation equal to the standard deviation of the data from each target variable. Different levels of regularization (*f*) were obtained for SRR by linearly spacing α values on a log_10_ scale from 10^−4^ to 10^5^ and for FRR by linearly spacing fractions from 0.05 to 1 in increments of 0.05.

Two versions of SRR were implemented and evaluated. The first version (naive) involves a separate matrix pseudo-inversion for each hyperparameter setting desired. The second version (rotation-based) involves using the SVD decomposition method described above (see Section Background and theory, specifically equation 14).

All simulations were run on an Intel Xeon E5-2683 2.10 GHz (32-core) workstation with 128 GB of RAM, a 64-bit Linux operating system, and MATLAB 8.3 (R2014a). Execution time was logged for model fitting procedures only and did not include generation of the design matrix or the data. Likewise, memory requirements were recorded in terms of additional memory usage during the course of model fitting (i.e., zero memory usage corresponds to the total memory usage just prior to the start of model fitting). Benchmarking results were averaged across 15 independent simulations to reduce incidental variability.

The code that implements these benchmarks is available in the “examples” folder of the software.

### Brain magnetic resonance imaging data

Brain functional magnetic resonance imaging (fMRI) data were collected as part of the Natural Scenes Dataset (http://naturalscenesdataset.org). Data were acquired in a 7T MRI instrument, at a spatial resolution of 1.8 mm and a temporal resolution of 1.6 seconds and using a matrix size of [81 104 83]. This yielded a total of 783,432 voxels. Over the course of 40 separate scan sessions, a neurologically healthy participant viewed 10,000 distinct images (three presentations per image) while fixating a small dot placed at the center of the images. The images were 8.4° × 8.4° in size. Each image was presented for three seconds and was followed by a one-second gap. Standard pre-processing steps were applied to the fMRI data to remove artifacts due to head motion and other confounding factors. To deal with session-wise nonstationarities, response amplitudes of each voxel were *z*-scored within each scan session. Responses were then concatenated across sessions and averaged across trials of the same image, and then a final *z*-scoring of each voxel’s responses was performed. The participant provided informed consent and the experimental protocol was approved by the University of Minnesota Institutional Review Board. For the purposes of the example demonstrated here, only the first 37 of the 40 scan sessions are provided (data are being held out for a prediction challenge), yielding a total of 9,841 distinct images.

A regression model was used to predict the response observed from a voxel in terms of local contrast present in the stimulus image. In the model, the stimulus image is pre-processed by taking the original color image (425 pixels × 425 pixels × 3 RGB channels), converting the image to grayscale, gridding the image into 25 × 25 regions, and then computing the standard deviation of luminance values within each grid region . This produced 625 predictors, each of which was then *z*-scored. The design matrix *X* has dimensionality 9,841 images × 625 stimulus regions, while *Y* has dimensionality 9,841 images × 783,432 voxels.

Cross-validation was carried out using a 80/20 training/testing split. For SRR, we evaluated a grid of alpha values that included 0 and ranged from 10^−4^ to 10^5.5^ in increments of 0.5 log_10_ units. For fractional ridge regression, we evaluated a range of fractions from 0 to 1 in increments of 0.05. Cross-validation performance was quantified in terms of variance explained on the test set using the coefficient of determination (*R*^2^).

The code that implements these analyses is available in the “examples” folder of the software.

## Results

### Fractional ridge regression achieves the desired outcomes

In simulations, we demonstrate that the FRR algorithm accurately produces the desired fractions γ (Fig. 2A and B second row, right column in each). We compare the results of FRR to results of SRR, in which α values were selected using a common heuristic (log-spaced values spanning a large range). For the SRR approach, we find that the fractional L2-norm is very small and virtually indistinguishable for large values of α, and is very similar to the OLS solution (fractional L2-norm ∼1) for several small values of α (Fig. 2A and B second row, left column). In addition, cross-validation accuracy is indistinguishable for many of the values of α evaluated in SRR. Only very few values of α produce cross-validated *R*^2^ values that are similar to the value provided by the best α (Fig. 2A and B first row, left column).

The SRR results can also be re-represented using effective degrees of freedom (DOF; Fig. 2A and B first row, middle column): several values of α result in essentially the same number of DOF, because these values are either much larger than the largest singular value or much smaller than the smallest singular value of *X*. In contrast to SRR, FRR produces a nicely behaved range of cross-validated *R*^2^ values and dense sampling around the peak *R*^2^.

Another line of evidence highlighting the diversity of the solutions provided by FRR is given by inspecting coefficient paths: in the log-spaced case, coefficients start very close to 0 (for high α) and rapidly increase (for lower α). Even when re-represented using DOF, the coefficient paths exhibit some redundancy. In contrast, FRR provides more gradual change in the coefficient paths, indicating that this approach explores the space of possible coefficient configurations more uniformly. Taken together, these analyses demonstrate that FRR provides a more useful range of regularization levels than SRR.

### FRR is computationally efficient

A question of relevance to potential users of FRR is whether using the method incurs significant computational cost. We compare FRR to two alternative approaches. The first approach is a naive implementation of the matrix inversion specified in equation 3, in which the Moore-Penrose pseudo-inverse (implemented as `pinv` in Matlab and `numpy.linalg.pinv` in Python) is performed independently for each setting of hyperparameter α. The second approach takes advantage of the computational expedience of the SVD-based approach: instead of a matrix inversion for each α value, a single SVD is performed, a transformation (rotation) is applied to the data, and different values of α are plugged into equation 14 to compute the regression coefficients. This approach comprises a subset of the operations taken in FRR. Therefore, it represents a lower bound in terms of computational requirements.

**Figure 3:**
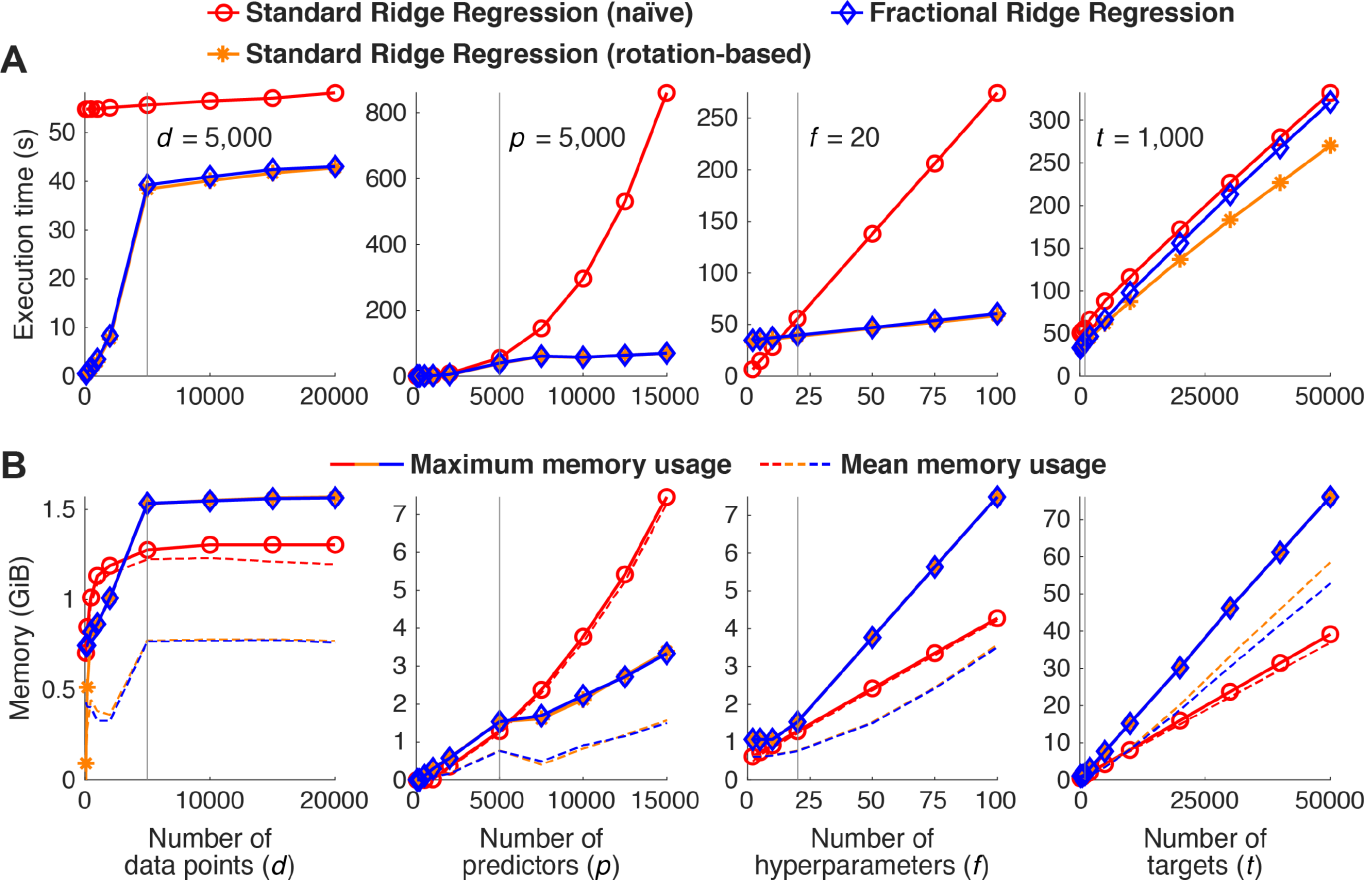
Computational efficiency. We benchmarked different methods for performing ridge regression: (1) a naive implementation of standard ridge regression (involving log-spaced α values) in which matrix inversion is performed for each α value, (2) an implementation of standard ridge regression in which solutions are computed in a rotated space based on singular value decomposition of the design matrix, and (3) the FRR method. Starting from a base case (*d* = 5,000, *p* = 5,000, *f* = 20, *b* = 1,000; parameter settings marked by vertical lines), we systematically manipulated *d, p, f*, and *t* (columns 1–4, respectively). (A) Execution time. The execution time of each method is shown in seconds. (B) Memory usage. The maximum memory usage of each method is shown as a solid line, whereas the time-averaged memory usage is shown as a dotted line. Overall, FRR is fast and has relatively modest memory requirements.

Through systematic exploration of different problem sizes, we find that FRR performs quite favorably. FRR differs from the rotation-based approach only slightly with respect to execution time scaling in the number of data points (Fig. 3A, left column), in the number of parameters (Fig. 3A, right column), and in *f*, the number of hyperparameter values considered (Fig. 3A, third column ). The naive matrix-inversion approach is faster than both SVD-based approaches (FRR and rotation-based) for *f* < 20 but rapidly becomes much more costly for values >20. This approach also scales rather poorly for *p* > 5,000.

In terms of memory consumption, the mean and maximum memory usage are similar for FRR and the naive and rotation-based SRR solutions. These results suggest that for each of these approaches, the matrix inversion (for the naive implementation of SRR) or the SVD (for FRR and the rotation-based SRR) represents the main computational bottleneck. Importantly, despite the fact that FRR uses additional gridding and interpolation steps, it does not perform substantially worse than either of the other approaches.

### Application of FRR to real-world data

**Figure 4:**
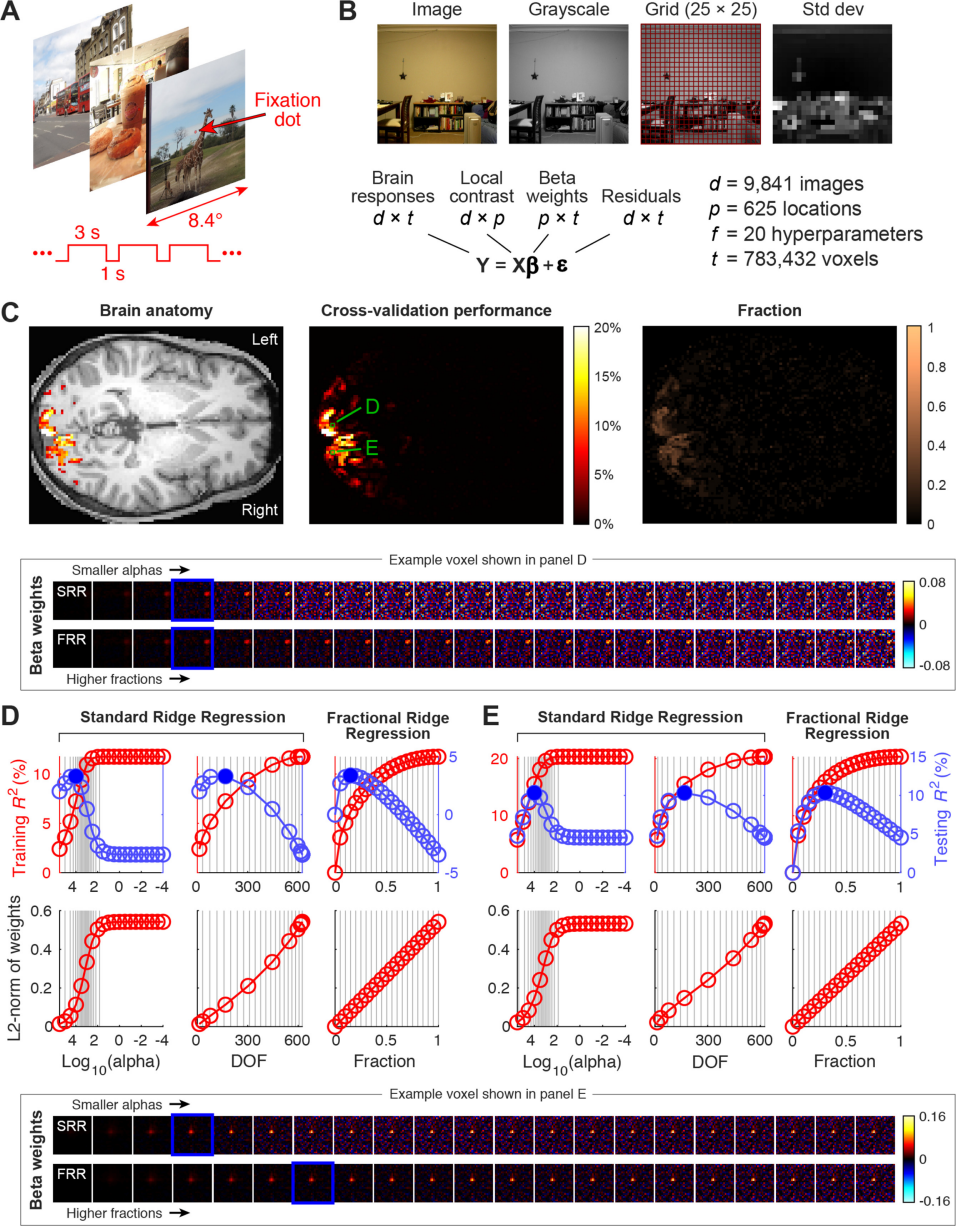
Demonstration on real-world data. (A) Visual fMRI experiment. Functional MRI measurements of brain activity were collected from a human participant while s/he viewed a series of natural images. (B) Model of brain activity. Images were converted to grayscale and gridded, and then the standard deviation of luminance values within each grid element was calculated. This produced measures of local contrast. Brain responses at every voxel were modeled using a weighted sum of local contrast. (C) Results obtained using FRR. Cross-validated performance (variance explained) achieved by the model is shown for an axial brain slice (middle). These results are thresholded at 5% and superimposed on an image of brain anatomy for reference (left). The fraction (γ) corresponding to the best cross-validation performance is also shown (right). (D) Detailed results for 1 voxel (see green squares in panel C). The main plots that depict training and testing performance and L2-norm are in the same format as Fig. 1. The inset illustrates coefficient solutions for different regularization levels. The blue box highlights the regularization level producing highest cross-validation performance. (E) Detailed results for a second voxel. Same format as panel D.

To demonstrate the practical utility of FRR, we explore its application in a specific scientific use-case. Data from an fMRI experiment were analyzed with FRR, and the results of this analysis were compared to an SRR approach where α values are selected using a log-spaced heuristic. Different parts of the brain process different types of information, and a large swath of the cerebral cortex is known to respond to visual stimulation. Experiments that combine fMRI with computational analysis provide detailed information about the responses of different parts of the brain [18]. In the experiments analyzed here, a series of images are shown (Fig.   4A) and the blood oxygenation level–dependent (BOLD) signal is recorded in a sampling grid of voxels throughout the brain . In the cerebral cortex, each voxel contains hundreds of thousands of neurons. If these neurons respond vigorously to the visual stimulus presented, the metabolic demand for oxygen in that part of cortex will drive a transient increase in oxygenated blood in that region, and the BOLD response will increase. Thus, a model of the BOLD response tells us about the selective responses of neurons in each voxel in cortex.

Because neurons in parts of the cerebral cortex that respond to visual stimuli are known to be particularly sensitive to local contrast, we model responses with respect to the standard deviation of luminance in each region of the image, rather than the luminance values themselves (Fig. 4B). In the model, *Y* contains brain responses where each target (column) represents the responses in a single voxel. Each row contains the response of all voxels to a particular image. The design matrix *X* contains the local contrast in every region of the image, for every image. This means that the coefficients β represent weights on the stimulus image and indicate each voxel’s spatial selectivity—i.e., the part of the image to which the voxel responds [19]. Therefore, one way to visualize $\hat{\beta }$ is to organize it according to the 2D layout of the image (Fig. 4C and D, bottom 2 rows).

Using FRR, we fit the model to voxel responses, and find robust model performance in the posterior part of the brain where visual cortex resides (left part of the horizontal slice presented in the top row of Fig. 4C). The performance of the model can be observed in either the cross-validated *R*^2^ values (Fig. 4C, top row, left and middle panels) or the value of γ corresponding to the best cross-validated *R*^2^ (top row, right panel). The γ values corresponding to best performance provide additional information about the differences between different targets, and an additional interpretation of the data. For example, we can focus on the two voxels highlighted in the middle panel of the top row in Fig.   4C. One voxel, whose characteristics are further broken down in Fig. 4D, has lower cross-validated $R^2 = 4\%$ and requires stronger relative regularization (γ = 0.15). The spatial selectivity of this voxel’s responses becomes very noisy at large γ values and in these values *R*^2^ approaches 0. On the other hand, the voxel in Fig. 4E has a higher best γ = 0.35 and a higher cross-validated $R^2=13\%$. Moreover, this voxel appears more robust with higher values of γ producing less spatially noisy results. The map of *R*^2^ and γ illustrated in Fig. 4C also shows that these trends hold more generally: voxels with more accurate models require less relative regularization. This demonstrates the additional interpretable information provided by the best γ values in individual targets and by inspecting spatial maps of these best γ values.

## Discussion

The main theoretical contribution of this work is a novel approach to hyperparameter specification in ridge regression. Instead of the standard approach in which a heuristic range of values for hyperparameter α are evaluated for their accuracy, the FRR approach focuses on achieving specific fractions for the L2-norms of the solutions relative to the L2-norm of the unregularized solution. In a sense, this is exactly in line with the original spirit of ridge regression, which places a penalty on the L2-norm of the solution. The main practical contribution of this work is the design and implementation of an efficient algorithm to solve FRR and validation of this algorithm on simulated and empirical data. Note that the FRR algorithm can be viewed as a method for finding appropriate α values that are adapted to the data such that they span the range of possible regularization strengths. Thus, it is fundamentally still a method that solves the SRR problem.

We emphasize that in theory, FRR and SRR are not expected to give different solutions to the linear regression problem. However, in practice, the solutions may very well differ and this will depend on the heuristic set of α values used in the SRR approach. Fractional ridge regression provides a method to automatically ensure proper setting of α values. Note that in the examples of SRR that we presented (e.g., Figs 2 and 4), well-selected heuristic ranges of α values were used. This is done deliberately because poor ranges of α values would have resulted in examples that are not very informative for this article. However, in everyday practice, a user of the SRR approach might inadvertently use an inappropriate range of α values and obtain poor results. Overall, we suggest that FRR can serve as a default approach to solving ridge regression.

### The benefits of FRR

1. **Theoretically motivated and principled**. The results demonstrate that the theoretical motivation described in the Methods holds in practice. Our implementation of FRR produces ridge regression solutions that have predictable and tuneable fractional L2-norm.
2. **Statistically efficient**. Each fraction level returned by FRR produces β values that are distinctly different. This avoids the common pitfall in the log-spaced approach whereby computation is wasted on several values of α that all over-regularize or under-regularize. When used with a range of γ values from 0 to 1, the solution that minimizes cross-validation error is guaranteed to exist within this range (although it may lie in between two of the obtained solutions).
3. **Computationally efficient**. We show that our implementation of FRR requires memory and computational time that are comparable to a naive ridge regression approach and to an approach that uses SVD but relies on preset α values. SVD-based approaches (including FRR) scale linearly in *f*, with compute-time scaling better than naive RR in the *f* > 20 regime. In practice, we have found that *f* = 20 evenly distributed values between 0 and 1 provide sufficient coverage for many problems. But the linear scaling implies that sampling more finely would not be limiting in cases where additional precision is needed.
4. **Interpretable**. FRR uses γ values that represent scaling relative to the L2-norm of the OLS solution. This allows FRR results to be compared across different targets within a dataset. This is exemplified in the results from an fMRI experiment that are interpreted in light of both cross-validated *R*^2^ and the optimal γ that leads to the best cross-validated *R*^2^. Moreover, regularization in different datasets and for different models (e.g., different settings of *X*) can be compared to each other as being stronger or weaker. The optimal regularization level can be informative regarding the signal-to-noise ratio of a particular target or about the level of collinearity of the design matrix (which both influence the optimal level of regularization). FRR increases the interpretability of ridge regression because instead of an unscaled, relatively inscrutable value of α, we receive a scaled, relatively interpretable value. Based on a recently proposed framework for interpretability in machine learning methods [20], we believe that this kind of advance improves the descriptive accuracy of ridge regression.
5. **Automatic**. Machine learning algorithms focus on automated inferences, but many machine learning algorithms still require substantial manual tuning. For example, if the range of α values used is not sufficient, users of ridge regression may be forced to explore other values. This is impractical in cases in which thousands of targets are analyzed and multiple models are evaluated. Thus, FRR contributes to the growing field of methods that aim to automate machine learning [21,22]. These methods all aim to remove the burden of manual inspection and tuning of machine learning. A major benefit of FRR is therefore practical in nature: Because FRR spans the dynamic range of effects that ridge regression can provide, using FRR guarantees that the time taken to explore hyperparameter values is well spent. Moreover, the user does not have to spend time speculating what α values might be appropriate for a given problem (e.g., is 10^4^ high enough?).
6. **Implemented in usable open-source software**. We provide code that is well documented, thoroughly tested, and easy to use (see project home page: https://nrdg.github.io/fracridge/). The software is available in two popular statistical programming languages: MATLAB and Python. The Python implementation provides an object-oriented interface that complies with the popular Scikit-Learn library [12,13].

### Using FRR in practice

To select the level of regularization to apply in practice, users of FRR will likely use cross-validation. An open question is how to aggregate the results of FRR over multiple cross-validation splits. This is a general issue for any analysis that uses cross-validation to set hyperparameters. Nevertheless, here we provide some ideas for how users can apply FRR in practice: (i) one could determine the optimal fraction using cross-validation on a single training/testing split (e.g., 80/20) and obtain a single model solution and a corresponding optimal fraction; (ii) one could determine the optimal fraction using cross-validation on a single training/testing split and then adopt that fraction for solving the regression on the full dataset, with the understanding that this may yield a slightly over-regularized solution; or (iii) one could determine the optimal fraction in different cross-validation splits of the data (e.g., *n*-fold cross-validation) and then average the determined fraction across the splits and average the estimated regression weights across the splits.

FRR is naturally integrated into a cross-validation framework where solutions reflecting different fractional lengths are obtained for a given set of data and evaluated for their predictive performance on held-out data. In the Python version of our software, this is implemented through an object that automatically performs a grid search to find the best value of γ among user-provided values. An alternative to performing cross-validation is the technique of generalized cross-validation (GCV). In GCV, for a given α value, matrix operations are used to efficiently estimate cross-validation performance without actually having to perform cross-validation [23]. It might be possible to combine the insights of FRR (e.g., the identification of interpretable and appropriate α values) with GCV.

### Limitations

One limitation of FRR is that a heuristic approach is used within the algorithm to generate the grid of α values used for interpolation (see Methods for details). Nonetheless, the interpolation results are quite accurate, and costly computations are carried out only for final desired α values. Another limitation is that the α value that corresponds to a specific γ may be different for different targets and models. If there are theoretical reasons to retain the same α across targets and models, the FRR approach is not appropriate. But this would rarely be the case because α values are usually not directly interpretable. Alternatively, FRR can be used to estimate values of α on one sample of the data (or for one model) and these values of α can then be used in all of the data (or all models).

Finally, the FRR approach is limited to ridge regression and does not generalize easily to other regularization approaches. The Lasso [24] provides regression solutions that balance least-squares minimization with the L1-norm of the coefficients rather than the L2-norm of the coefficients. The Lasso approach has several benefits, including results that are sparser and potentially easier to interpret. Similarly, Elastic Net [25] uses both L1- and L2-regularization, potentially offering more accurate solutions. But because the computational implementation of these approaches differs quite substantially from ridge regression, the approach presented in this article does not easily translate to these methods. Moreover, while these methods allow regularization with a non-negativity constraint on the coefficients, this constraint is not easily incorporated into L2-regularization. On the other hand, a major challenge that arises in L1-regularization is computational time: most algorithms operate on one target at a time and incur substantial computational costs, and scaling such algorithms to the thousands of targets in large-scale datasets may be difficult.

### Future extensions

An important extension of the present work would be an implementation of these ideas in additional statistical programming languages, such as the R programming language, which is popular for use in statistical analysis of data from many different domains. One of the most important tools for regularized regression is the GLMNET software package, which was originally implemented in the R programming language [26] and has implementations in matlab [27] and Python [28]. The software also provides tools for analysis and visualization of coefficient paths and of the effects of regularization on cross-validated error. The R GLMNET vignette [29] demonstrates the use of these tools. In addition to identifying the α value that minimizes cross-validation error, GLMNET also identifies the α that gives the most regularized model such that the cross-validated error is within one standard error of the minimum cross-validated error. This approach acknowledges that there is some error in selecting α and chooses to err on the side of a more parsimonious model [5]. Future extensions of FRR could implement this heuristic.

## Availability of Source Code and Requirements

- Project name: Fractional Ridge Regression
- Project home page:  http://github.com/nrdg/fracridge
- Operating system(s): Platform independent
- Programming language: Python or MATLAB
- License: 3-clause BSD
- Biotools URL: https://bio.tools/fracridge
- RRID:SCR_019045

## Data Availability

Code and data to reproduce the figures in this manuscript are available under a CC-BY license through GigaDB [30].

## Consent for Publication

Consent to publish has been obtained from the fMRI participant as part of the informed consent procedure (see Methods).

## Abbreviations

API: Application Programming Interface; BOLD: blood oxygenation level dependent; DOF: degrees of freedom; fMRI: functional magnetic resonance imaging; FRR: fractional ridge regression; GCV: generalized cross-validation; OLS: ordinary least squares; OSI: Open Source Initiative; RAM: random access memory; SRR: standard ridge regression; SVD: singular value decomposition.

## Competing Interests

The authors declare that they have no competing interests.

## Funding

AR was funded through a grant from the Gordon & Betty Moore Foundation and the Alfred P. Sloan Foundation to the University of Washington eScience Institute, through NIH grants 1RF1MH121868-01 from the National Institute for Mental Health (PI: AR) and 5R01EB027585-02 from the National Institute for Biomedical Imaging and Bioengineering (PI: Eleftherios Garyfallidis, Indiana University) and through NSF grants 1934292 (PI: Magda Balazinska, University of Washington). KK was supported by NIH P41 EB015894. Collection of MRI data was supported by NSF IIS-1822683, NSF IIS-1822929, NIH S10 RR026783, and the W.M. Keck Foundation.

## Author Contributions

Both authors conceived the algorithm. Both authors implemented software. K.K. conducted simulations and data analysis. Both authors wrote the manuscript.

## Acknowledgments

The authors thank Noah Simon for helpful discussions and Noah Benson for comments on the manuscript.

## Supplementary Material

giaa133_GIGA-D-20-00231_Original_Submission

giaa133_GIGA-D-20-00231_Revision_1

giaa133_GIGA-D-20-00231_Revision_2

giaa133_Response_to_Reviewer_Comments_Original_Submission

giaa133_Response_to_Reviewer_Comments_Revision_1

giaa133_Reviewer_1_Report_Original_Submission Siyuan Gao -- 9/1/2020 Reviewed

giaa133_Reviewer_1_Report_Revision_1 Siyuan Gao -- 10/7/2020 Reviewed

giaa133_Reviewer_2_Report_Original_Submission Tom DuprÃ© la Tour -- 9/2/2020 Reviewed

giaa133_Reviewer_2_Report_Revision_1 Tom DuprÃ© la Tour -- 10/5/2020 Reviewed
