# Supplementary material for: Fractional ridge regression: a fast, interpretable reparameterization of ridge regression
Source: Gigascience. 2020 Nov 30;9(12):giaa133. doi: 10.1093/gigascience/giaa133 (PMC7702219; doi:10.1093/gigascience/giaa133)
Supplement: giaa133_Response_to_Reviewer_Comments_Original_Submission [file giaa133_response_to_reviewer_comments_original_submission.pdf]

Author's Response To Reviewer Comments

Close

September 28th, 2020

Dear Editor,

Thank you for your consideration of our manuscript GIGA-D-20-00231.

We are pleased with the high-quality comments and suggestions from the reviewers. We have revised the manuscript to address the concerns raised. In addition, as requested, we have registered our software tool in the bio.tools and SciCrunch.org databases, and we include the RRID in the revised manuscript.

Changes to the manuscript are marked in red text and include the following:

1. Discussion about the choice of alpha ranges used for the examples in the paper. Both reviewers commented on this aspect of the paper (Points 1.3, 2.12), and the revised manuscript now clarifies why this was done and what inferences the reader should make (see p. 8-9, lines 565-578).

2. Additional code implementation features and data documentation. We have revised the provided code and data to implement the suggestions of the reviewers (Points 2.7, 2.8, 2.15). Please see the online materials for these changes.

3.Other clarifications and discussion points. We have revised the manuscript to address a number of conceptual points raised by the reviewers.

Please find below a point-by-point response to the reviewers' comments.

Thank you for your consideration,

Ariel Rokem
Research Assistant Professor in Psychology
University of Washington

Kendrick Kay
Assistant Professor in Radiology
University of Minnesota, Twin Cities

Response to Reviewer 1

Point 1.1. “Reviewer #1: In this paper, the authors reparameterized ridge regression so that the penalty parameter can be more efficiently tuned and is more interpretable. In specific, the substitute hyper parameter gamma can be interpreted as the fraction of shrinkage applied to the overall L2-norm of the solution. By using this approach, it avoids choosing the upper and lower limit of the original alpha penalty. Moreover, the regression coefficients and model performance also change more distinctly by changing the gamma parameter. Overall, this is an applicable and seems efficient approach for a lot of neuroimaging or other studies that used ridge regression. The manuscript was also written clearly and easy to follow. My comments are as follows,”

We thank the reviewer for their summary and evaluation of our work.

Point 1.2. “1. From my understanding, the biggest problem this reparametrization solves is to choose a more appropriate parameter grid for cross-validation. With that being said, in the standard ridge regression the author compared, the maximum and minimum alpha values all seem a bit too extreme and they are fixed. So a lot of computations are wasted on the alphas close to the two ends. Can the authors discuss if it is possible to choose the alpha range sensitive to the data (e.g., adjust based on the singular value of design matrix) and if that can leads to closer performance of the standard ridge to the fractional ridge?”

The reviewer here points out one of the limitations of the standard approach to ridge regression: the user typically chooses a heuristic range of alpha values, and this range could risk wasting a lot of computation.

Indeed, this limitation is exactly one of the main issues that our fractional ridge regression approach seeks to remedy. We show in our paper how the fractional ridge regression approach is able to provide well-chosen alpha values (as suggested by the reviewer).

Action: The revised manuscript further highlights and explains how fractional ridge regression judiciously determines the alpha range and the sampling of alphas within that range (p. 8, lines 559-564). This should help clarify how fractional ridge regression improves on the standard approach.

Point 1.3. “2. Although the model performance change more rapidly and consistently with fractional ridge, the optimal performance can also be reached with the standard ridge in most of the experiment plots shown. Can the author comment on how often can the fractional ridge find better parameters that are totally skipped by standard ridge regression? For example, is figure 4c look different with the standard ridge regression that uses the fixed alpha grid?”

The reviewer correctly points out that it is possible that optimal performance can be reached by both standard ridge regression and fractional ridge regression: if one samples infinitely densely spaced alpha values, one is guaranteed to find the maximum. However, the critical point is that in practice, standard ridge regression may miss optimal performance depending on incidental choices made in the heuristic range of alpha values used.

Action: The revised manuscript now clarifies that, in theory, fractional ridge regression and standard ridge regression are not expected to give different solutions to the ridge regression problem (see p. 8-9, lines 565-578). However, in practice, the solutions may very well differ and this will depend on the heuristic set of alpha values used in the standard ridge regression approach. What fractional ridge regression provides is a method to automatically ensure proper setting of alpha values.

Furthermore, the revised manuscript now explicitly points out (see p. 9, lines 571-575) that the examples we provide in the manuscript involve a well-selected heuristic range of alpha values. This is done deliberately, as a poor range of alpha values would not be very informative to show in the manuscript. However, in practice, a user of the standard ridge regression approach might inadvertently use an inappropriate range of alpha values and obtain poor results.

Point 1.4. “3. In the experiments the authors performed, the number of predictors are similar to or smaller than the number of data points. In neuroimaging studies especially functional connectivity studies, often times the number of predictors is a lot more than the number of data points. Can the authors comment on if fractional ridge is still efficient when p>>d?”

Yes, there is no specific efficiency loss if p>>d; the computational requirements are comparable whether d>p or p>d. Notice that in the benchmarking figure, we confirm that cases where p >> d are still handled gracefully by our algorithm (see Figure 3, second column).

Action: The revised manuscript now discusses the computational issues regarding the size of d and size of p (see p. 5, lines 259-267).

Response to Reviewer 2

Point 2.1. “Reviewer #2: This paper describes a reparametrization of the regularization coefficient in ridge regression, using a hyperparameter gamma (in [0, 1]) instead of the standard hyperparameter alpha (in [0, inf]). This new hyperparameter space depends on the training data set, and provides a more principled grid of hyperparameter for grid-search over cross-validation.”

We thank the reviewer for their summary and evaluation of our work.

Point 2.2. “Method // ------ // Strengths:

- The proposed reparametrization is sound and well presented.

- The method is indeed a more principled way to define a grid of hyperparameter.

- The mathematical description is correct.

- It is sufficiently described to allow reproducibility.”

We acknowledge these strengths.

Point 2.3. “Limitations: // - It is not clear how to aggregate the results of FRR over multiple cross-validation splits. Indeed, since the gamma parametrization depends on the data, using different splits leads to different grids of corresponding alphas, which might not be straightforward to aggregate. The authors should either mention this limitation, or describe a way to address it.”

Here the reviewer points out that the gamma parameterization might be tricky to combine or aggregate across cross-validation splits. We agree with this point. However, we would like to highlight the fact that this is a general issue that affects any hyperparameterization, including the “alpha” parameterization in standard ridge regression. Consider, for example, the case of having access to 1,000 data points in a given regression problem. The optimal alpha determined for this scenario (for example, evaluated on a separate set of data) will likely differ from the optimal alpha if there had instead been access to 10,000 data points. Specifically, one would expect the optimal alpha to be smaller for the larger dataset since with more data points, one is likely better able to estimate the regression weights using unbiased estimation, and so less regularization of the weights is required to achieve good performance. Thus, even in standard ridge regression, one must somehow combine alpha results across splits.

We acknowledge that the corresponding grid of alphas (for a fixed set of fractions) might be different for different data splits in fractional ridge regression. However, we don’t see any reason why one should prefer to average alphas as opposed to just averaging fractions. Indeed, one might speculate that fractions might constitute a more stable feature of a regression problem, but a detailed investigation would be necessary to carefully assess that issue.

Action: The revised manuscript and added a section to the Discussion that now addresses the issue raised by the reviewer and, as requested by the reviewer, we additionally provide some ideas of how users can aggregate fractional ridge regression results across cross-validation splits (see Discussion p. 10, lines 650-680).

Point 2.4. “- The gamma parametrization is different on each target, so the algorithm cannot leverage broadcasting over targets, and needs to perform a slow loop over targets. This should be mentioned as a limitation.”

The reviewer is correct that fractional ridge regression necessitates looping over targets since in order to achieve a desired fractional regularization level, one is forced to implement a different parameterization for each target. Note that we find that this does not result in major performance degradation, since the bulk of the computational cost in ridge regression is taken by the SVD operation. See 2.10 below as well.

Action: The revised manuscript now acknowledges the issue raised by the reviewer (see Methods p. 4, lines 229-232).

Point 2.5. “Implementation // -------------- // Strengths:

- The software implementation is available both in MATLAB and Python, which is a great effort for dissemination of this work.

- The code is well structured, tested, and documented.

- It is publicly available on GitHub, and straightforward to install through PyPI.

- The software license is compliant with the Open Source Initiative.

- The Python code contains an estimator compatible with scikit-learn.

- The MATLAB code to produce all figures is fully available on the public repository."

We acknowledge these strengths.

Point 2.6. "Suggestions:

- FracRidge could be tested through the scikit-learn estimator checks. (https://scikit-learn.org/stable/modules/generated/sklearn.utils.estimator_checks.check_estimator.html)"

The unit tests now include a function `test_FracRidge_estimator`, which checks the estimator described in the previous version of the manuscript, as well as an additional estimator implemented in the revised manuscript. (https://github.com/nrdg/fracridge/blob/master/fracridge/tests/test_fracridge.py#L60)

Action: We have also added more details about this in the Methods description of the Scikit Learn-compatible API (p 5, line 283-285).

Point 2.7. "- FracRidge could be improved to provide a direct replacement to scikit-learn's Ridge estimator. It is currently not the case since FracRidge.predict returns a separate prediction for each value of the gamma hyperparameter, while Ridge.predict returns only one prediction.

- Actually, since FRR is optimizing the grid of hyperparameter for cross-validation, it would make more sense to make FracRidge a direct replacement to scikit-learn's RidgeCV estimator. (It is however not straightforward to aggregate the hyperparameter selection of FRR over multiple cross-validation splits, as mentioned earlier.)"

We thank the reviewer for these insightful observations. Much of the efficiency of the current implementation comes from performing a single SVD on the design matrix. While this means that the estimator we implemented is not a direct replacement of the Ridge estimator, to our understanding, this use-case is fully supported by the Scikit Learn API (see https://scikit-learn.org/stable/modules/generated/sklearn.multioutput.MultiOutputRegressor.html). Nevertheless, the suggestion to implement a cross-validation estimator is a good one and we have implemented an additional `FracRidgeRegressorCV` class that uses a grid-search approach to find the best gamma value for a dataset, and also provides the different alpha values that are a consequence of this value. Aggregation is done over gamma, rather than alpha, so this is not a limiting issue (see also our comments to point 2.3 above).

Action: We have augmented the Python implementation with a new estimator. This estimator is described on p. 5, line 285-287. We also provide an example of the use of this estimator in Figure 1. We note that the RidgeCV estimator involves the notion of generalized cross-validation; since this concept is related to the paper, we now discuss fractional ridge regression in the context of GCV (see p. 10, lines 674-680).

Point 2.8. "- The documentation website is rather limited, the authors might want to add a few more small examples.`"

Action: We have overhauled the documentation of the software, including a user guide and detailed API

documentation. As suggested, we have also added several examples, both small (embedded in the API documentation), and large (as a separate example gallery).

Point 2.9. “Simulations // ----------- // Strengths:

- The simulation settings are reasonable, and sufficiently describe for reproducibility.

- Figure 2 does support the claim that FRR automatically span the relevant range of regularization.

- Figure 3 does support the claim that FRR is as fast as the "rotation-based" baseline (except for large numbers of targets).”

We acknowledge these strengths.

Point 2.10. “Limitations:

- The "rotation-based" baseline in Figure 3 could be improved by using broadcasting over targets. This should not change most of the results, except the scaling over large numbers of targets (top right), where I expect the "rotation-based" baseline to be even better.”

The reviewer here suggests the valid point that speed improvements might be achieved if the implementation of the standard ridge regression approach (rotation-based) used broadcasting over targets.

When we were optimizing the code architecture and design, we did indeed experiment with different design choices. In our hands, it appeared that there is a trade-off between speed and memory usage. To be specific, consider the following lines in the MATLAB implementation:
https://github.com/nrdg/fracridge/blob/master/matlab/fracridge.m#L411-L413

The choice boils down to either (1) looping over targets and in each iteration using repmat to expand ynew(:,ii) or (2) using repmat to expand both sc and ynew and performing a single element-wise matrix multiplication. The existing code chooses the first path. We found that the second path incurs substantial memory overhead (which is especially problematic for big-data applications, like the ones explored in the benchmarking figure), with very little speed improvements. Hence, we believe that the current implementation is a reasonable choice for the paper.

Point 2.11. “Application to fMRI data // ------------------------ // Strengths:

- The data collection and preprocessing is well described.

- The data and model used are an appropriate application for the proposed method.

- The results in Figure 4C are reasonable, and show the soundness of the data and model.

- Figure 4D and 4E show that FRR adapts the grid depending on the target, leading to better hyperparameter selection.”

We acknowledge these strengths.

Point 2.12. “Limitations:

- Figure 4 does not show that the FRR leads to better results in generalization. With a fine grid of hyperparameter, it is not clear if a finer selection of alpha (with FRR) leads to significant improvements in model generalization, or interpretation. Using a coarser grid for both SRR and FRR might better demonstrate the improvement of FRR compared to SRR. Using generalization performances in a separate test set (different from the validation set used for cross-validation) might be a good metric to show the improvement of FRR over SRR.”

Here the reviewer points out that the application example does not necessarily show evidence of better generalization/interpretation.

We agree with this point. We chose to use a relatively fine grid for the examples because we feel the paper would risk being misunderstood otherwise. (For example, if we chose a poor grid that missed the dynamic range, that might be construed as a “straw man” argument.) That being said, we agree that there is a conceptual point here that needs to be better explained by the manuscript.

Action: The revised manuscript now clarifies the important point that the ranges used for the examples in the paper are deliberately actually “good” ranges and are designed to explain the concepts of the paper, but that in practice, “bad” ranges are certainly possible and that is the problem that fractional ridge regression solves (see p. 9, lines 571-575).

Point 2.13. “- It is not clear how Figure 4 shows that FRR is more intepretable than SRR.”

Action: The revised manuscript now explains that FRR provides the gamma (fraction) estimated for each voxel and that this can be useful for the scientist to compare across voxels (see p. 8, lines 529-531).

Point 2.14. “Data // ---- // Strengths:”

- Preprocessed data is publicly available for reproducing Figure 4.

We acknowledge these strengths.

Point 2.15. “Suggestions:

- The data could be described in a README file stored with the data, listing the different arrays stored in each file, their shapes, and their contents.

- The data could also contain the preprocessed "contrast grid" (25 x 25 x 10000).”

Action: We have implemented these useful suggestions.

Point 2.16. “Minor comments // --------------

- The definition of the L2 norm (on beta) in the introduction is ambiguous

Action: We have disambiguated this by adding parentheses (p 1, line 22).

- Eq (1), not clear if y is single or multiple target.

Thank you for noticing this error on our part.

Action: We have now clarified this by designating capital Y to represent the entire dataset (multiple targets) and lower-case y represents a single target (p. 2, lines 85, 86).

- Eq (2), the transpose notation is never defined.

Action: We have clarified this notation (p. 2, line 94). We also now use the more commonly-used latex “intercal” notation.

- The "squared error" used as objective for OLS is never defined.

Action: We have now added a definition of this error (p. 2, line 96)

- unifying the notation of matrix dimensions would help ("Y is the (d, t) matrix", "dimensionality d by 1", "dim(U) = (d, p)")

Action: We have unified this to use the “dimensionality of d by 1” formulation everywhere.

- Eq (8), why use two notations for applying S^-2 and S? Why introduce the Hadamard product here ? (9) is more clear than (8).

Action: We have simplified this to use matrix multiplication notation instead of the Hadamard product. We also explain the difference between equation 9 (single coordinate i) and the previous equations (p. 3, line 122).

- "full regularization" is not defined.

This corresponds to a value of alpha (i.e. infinity) that would be high enough to reduce all of the coefficients to be equal to 0.

Action: We now explain this on p. 3, line 156.

- Eq (19), p should be placed just before the summations, to avoid interpreting it as a function or a probability.

Thanks! That’s a helpful suggestion.

Action: It turns out there was a slight error in this equation, so in our revision, this issue has gone away.

- (iv) The "FRR requirements" are not defined.

Action: This is now defined: it is the values of alpha required to achieve the requested gamma. We have simplified this sentence to make this clearer (p. 4, line 210).

- (iv) Do you have a proof for the fact that "the values of alpha are guaranteed to lie within a range that depends on the eigenvalues of X" ?

We did not intend to make a theoretical claim here. Rather, we are just restating the implications of

equation 18 in words.

Action: We have reworded this sentence to avoid confusion and now refer the reader back to equation 18, so this is clear (p. 4, lines 210-212).

- Figure 2: "vector length of solution" is not clear, what about changing to "vector norm of the solution"?

Action: We have changed Figures 2 and 4 to use the phrase “L2-norm” (consistent with the rest of the paper).

- Figure 4: the black arrows to the inset are confusing, since they point to two particular columns that do not correspond to anything. Moreover, the horizontal axis of the inset is not defined, and seem to be different for SRR and FRR.”

Action: We have implemented these useful suggestions: we have changed the figure design to get rid of the black arrows and to define the horizontal axis of the inset.

Close
