# Supplementary material for: Fractional ridge regression: a fast, interpretable reparameterization of ridge regression
Source: Gigascience. 2020 Nov 30;9(12):giaa133. doi: 10.1093/gigascience/giaa133 (PMC7702219; doi:10.1093/gigascience/giaa133)
Supplement: giaa133_Response_to_Reviewer_Comments_Revision_1 [file giaa133_response_to_reviewer_comments_revision_1.pdf]

Author's Response To Reviewer Comments

Close

Dear Dr. Nogoy,

Thank you for the positive assessment of our revision.

We have added the reference to the data in GigaDB, as marked up by you on the previous submission.

We have now also removed all of the colored markup, previously used to designate revisions from the first initial submission.

Best regards,

Ariel Rokem
Kendrick Kay

Close
