# Supplementary material for: Fractional ridge regression: a fast, interpretable reparameterization of ridge regression
Source: Gigascience. 2020 Nov 30;9(12):giaa133. doi: 10.1093/gigascience/giaa133 (PMC7702219; doi:10.1093/gigascience/giaa133)
Supplement: giaa133_Reviewer_1_Report_Original_Submission — Siyuan Gao -- 9/1/2020 Reviewed [file giaa133_reviewer_1_report_original_submission.pdf]

Reviewer Report

Title: Fractional ridge regression: a fast, interpretable reparameterization of ridge regression

Version: Original Submission **Date:** 9/1/2020

Reviewer name: Siyuan Gao

Reviewer Comments to Author:

In this paper, the authors reparameterized ridge regression so that the penalty parameter can be more efficiently tuned and is more interpretable. In specific, the substitute hyper parameter gamma can be interpreted as the fraction of shrinkage applied to the overall L2-norm of the solution. By using this approach, it avoids choosing the upper and lower limit of the original alpha penalty. Moreover, the regression coefficients and model performance also change more distinctly by changing the gamma parameter. Overall, this is an applicable and seems efficient approach for a lot of neuroimaging or other studies that used ridge regression. The manuscript was also written clearly and easy to follow. My comments are as follows,

1. From my understanding, the biggest problem this reparametrization solves is to choose a more appropriate parameter grid for cross-validation. With that being said, in the standard ridge regression the author compared, the maximum and minimum alpha values all seem a bit too extreme and they are fixed. So a lot of computations are wasted on the alphas close to the two ends. Can the authors discuss if it is possible to choose the alpha range sensitive to the data (e.g., adjust based on the singular value of design matrix) and if that can leads to closer performance of the standard ridge to the fractional ridge?
2. Although the model performance change more rapidly and consistently with fractional ridge, the optimal performance can also be reached with the standard ridge in most of the experiment plots shown. Can the author comment on how often can the fractional ridge find better parameters that are totally skipped by standard ridge regression? For example, is figure 4c look different with the standard ridge regression that uses the fixed alpha grid?
3. In the experiments the authors performed, the number of predictors are similar to or smaller than the number of data points. In neuroimaging studies especially functional connectivity studies, often times the number of predictors is a lot more than the number of data points. Can the authors comment on if fractional ridge is still efficient when p>>d?

Level of Interest

Please indicate how interesting you found the manuscript: Choose an item.

Quality of Written English

Please indicate the quality of language in the manuscript: Choose an item.

Declaration of Competing Interests

Please complete a declaration of competing interests, considering the following questions:

- Have you in the past five years received reimbursements, fees, funding, or salary from an organisation that may in any way gain or lose financially from the publication of this manuscript, either now or in the future?
- Do you hold any stocks or shares in an organisation that may in any way gain or lose financially from the publication of this manuscript, either now or in the future?
- Do you hold or are you currently applying for any patents relating to the content of the manuscript?
- Have you received reimbursements, fees, funding, or salary from an organization that holds or has applied for patents relating to the content of the manuscript?
- Do you have any other financial competing interests?
- Do you have any non-financial competing interests in relation to this paper?

If you can answer no to all of the above, write 'I declare that I have no competing interests' below. If your reply is yes to any, please give details below.

I declare that I have no competing interests

I agree to the open peer review policy of the journal. I understand that my name will be included on my report to the authors and, if the manuscript is accepted for publication, my named report including any attachments I upload will be posted on the website along with the authors' responses. I agree for my report to be made available under an Open Access Creative Commons CC-BY license (http://creativecommons.org/licenses/by/4.0/). I understand that any comments which I do not wish to be included in my named report can be included as confidential comments to the editors, which will not be published.

Choose an item.

To further support our reviewers, we have joined with Publons, where you can gain additional credit to further highlight your hard work (see: https://publons.com/journal/530/gigascience). On publication of this paper, your review will be automatically added to Publons, you can then choose whether or not to claim your Publons credit. I understand this statement.

Yes Choose an item.
