# Supplementary material for: Fractional ridge regression: a fast, interpretable reparameterization of ridge regression
Source: Gigascience. 2020 Nov 30;9(12):giaa133. doi: 10.1093/gigascience/giaa133 (PMC7702219; doi:10.1093/gigascience/giaa133)
Supplement: giaa133_Reviewer_2_Report_Original_Submission — Tom DuprÃ© la Tour -- 9/2/2020 Reviewed [file giaa133_reviewer_2_report_original_submission.pdf]

Reviewer Report

Title: Fractional ridge regression: a fast, interpretable reparameterization of ridge regression

Version: Original Submission **Date:** 9/2/2020

Reviewer name: Tom DuprÃ© la Tour

Reviewer Comments to Author:

This paper describes a reparametrization of the regularization coefficient in ridge regression, using a hyperparameter gamma (in [0, 1]) instead of the standard hyperparameter alpha (in [0, inf]). This new hyperparameter space depends on the training data set, and provides a more principled grid of hyperparameter for grid-search over cross-validation.

Method

Strengths:

- The proposed reparametrization is sound and well presented.
- The method is indeed a more principled way to define a grid of hyperparameter.
- The mathematical description is correct.
- It is sufficiently described to allow reproducibility.

Limitations:

- It is not clear how to aggregate the results of FRR over multiple cross-validation splits. Indeed, since the gamma parametrization depends on the data, using different splits leads to different grids of corresponding alphas, which might not be straightforward to aggregate. The authors should either mention this limitation, or describe a way to address it.
- The gamma parametrization is different on each target, so the algorithm cannot leverage broadcasting over targets, and needs to perform a slow loop over targets. This should be mentioned as a limitation.

Implementation

Strengths:

- The software implementation is available both in MATLAB and Python, which is a great effort for dissemination of this work.
- The code is well structured, tested, and documented.
- It is publicly available on GitHub, and straightforward to install through PyPI.
- The software license is compliant with the Open Source Initiative.
- The Python code contains an estimator compatible with scikit-learn.
- The MATLAB code to produce all figures is fully available on the public repository.

Suggestions:

- FracRidge could be tested through the scikit-learn estimator checks. (https://scikit-learn.org/stable/modules/generated/sklearn.utils.estimator_checks.check_estimator.html)
- FracRidge could be improved to provide a direct replacement to scikit-learn's Ridge estimator. It is currently not the case since FracRidge.predict returns a separate prediction for each value of the gamma

hyperparameter, while Ridge.predict returns only one prediction.
- Actually, since FRR is optimizing the grid of hyperparameter for cross-validation, it would make more sense to make FracRidge a direct replacement to scikit-learn's RidgeCV estimator. (It is however not straightforward to aggregate the hyperparameter selection of FRR over multiple cross-validation splits, as mentioned earlier.)
- The documentation website is rather limited, the authors might want to add a few more small examples.

Simulations

Strengths:
- The simulation settings are reasonable, and sufficiently describe for reproducibility.
- Figure 2 does support the claim that FRR automatically span the relevant range of regularization.
- Figure 3 does support the claim that FRR is as fast as the "rotation-based" baseline (except for large numbers of targets).

Limitations:
- The "rotation-based" baseline in Figure 3 could be improved by using broadcasting over targets. This should not change most of the results, except the scaling over large numbers of targets (top right), where I expect the "rotation-based" baseline to be even better.

Application to fMRI data

Strengths:
- The data collection and preprocessing is well described.
- The data and model used are an appropriate application for the proposed method.
- The results in Figure 4C are reasonable, and show the soundness of the data and model.
- Figure 4D and 4E show that FRR adapts the grid depending on the target, leading to better hyperparameter selection.

Limitations:
- Figure 4 does not show that the FRR leads to better results in generalization. With a fine grid of hyperparameter, it is not clear if a finer selection of alpha (with FRR) leads to significant improvements in model generalization, or interpretation. Using a coarser grid for both SRR and FRR might better demonstrate the improvement of FRR compared to SRR. Using generalization performances in a separate test set (different from the validation set used for cross-validation) might be a good metric to show the improvement of FRR over SRR.
- It is not clear how Figure 4 shows that FRR is more intepretable than SRR.

Data

Strengths:
- Preprocessed data is publicly available for reproducing Figure 4.

Suggestions:
- The data could be described in a README file stored with the data, listing the different arrays stored in each file, their shapes, and their contents.
- The data could also contain the preprocessed "contrast grid" (25 x 25 x 10000).

Minor comments

- The definition of the L2 norm (on beta) in the introduction is ambiguous.
- Eq (1), not clear if y is single or multiple target.
- Eq (2), the transpose notation is never defined.
- The "squared error" used as objective for OLS is never defined.
- unifying the notation of matrix dimensions would help ("Y is the (d, t) matrix", "dimensionality d by 1", "dim(U) = (d, p)")
- Eq (8), why use two notations for applying S^-2 and S? Why introduce the Hadamard product here ? (9) is more clear than (8).
- "full regularization" is not defined.
- Eq (19), p should be placed just before the summations, to avoid interpreting it as a function or a probability.
- (iv) The "FRR requirements" are not defined.
- (iv) Do you have a proof for the fact that "the values of alpha are guaranteed to lie within a range that depends on the eigenvalues of X" ?
- Figure 2: "vector length of solution" is not clear, what about changing to "vector norm of the solution"?
- Figure 4: the black arrows to the inset are confusing, since they point to two particular columns that do not correspond to anything. Moreover, the horizontal axis of the inset is not defined, and seem to be different for SRR and FRR.

Level of Interest

Please indicate how interesting you found the manuscript: Choose an item.

Quality of Written English

Please indicate the quality of language in the manuscript: Choose an item.

Declaration of Competing Interests

Please complete a declaration of competing interests, considering the following questions:

- Have you in the past five years received reimbursements, fees, funding, or salary from an organisation that may in any way gain or lose financially from the publication of this manuscript, either now or in the future?
- Do you hold any stocks or shares in an organisation that may in any way gain or lose financially from the publication of this manuscript, either now or in the future?
- Do you hold or are you currently applying for any patents relating to the content of the manuscript?

- Have you received reimbursements, fees, funding, or salary from an organization that holds or has applied for patents relating to the content of the manuscript?
- Do you have any other financial competing interests?
- Do you have any non-financial competing interests in relation to this paper?

If you can answer no to all of the above, write 'I declare that I have no competing interests' below. If your reply is yes to any, please give details below.

I declare that I have no competing interests.

I agree to the open peer review policy of the journal. I understand that my name will be included on my report to the authors and, if the manuscript is accepted for publication, my named report including any attachments I upload will be posted on the website along with the authors' responses. I agree for my report to be made available under an Open Access Creative Commons CC-BY license (http://creativecommons.org/licenses/by/4.0/). I understand that any comments which I do not wish to be included in my named report can be included as confidential comments to the editors, which will not be published.

Choose an item.

To further support our reviewers, we have joined with Publons, where you can gain additional credit to further highlight your hard work (see: https://publons.com/journal/530/gigascience). On publication of this paper, your review will be automatically added to Publons, you can then choose whether or not to claim your Publons credit. I understand this statement.

Yes Choose an item.
